# Long-term treatment with denosumab in patients with celiac disease and osteoporosis at high risk of fracture: a retrospective study

**DOI:** 10.1007/s11739-026-04360-8

**Published:** 2026-04-28

**Authors:** Jessica Pepe, Luciano Colangelo, Rachele Santori, Marco Occhiuto, Daniele Diacinti, Evaristo Ettorre, Giovambattista Desideri, Salvatore Minisola, Cristiana Cipriani

**Affiliations:** 1 Department of Medical and Cardiovascular Sciences, “Sapienza” University of Rome, Viale del Policlinico 155, 00161 Rome, Italy; 2 Department of Radiological Sciences, Oncology and Pathology, “Sapienza” University of Rome, Viale del Policlinico 155, 00161 Rome, Italy

**Keywords:** Osteoporosis, Celiac disease, Denosumab, Fractures

## Abstract

Patients with celiac disease have lower bone mineral density (BMD) and higher incidence of fractures compared to age- and sex-matched controls. There are no studies of denosumab, an antiresorptive drug, which is a fully human monoclonal antibody that binds the receptor activator of NFκB ligand (RANKL) in celiac patients with osteoporosis. The aim is to study the long-term effect of denosumab on BMD in celiac patients with osteoporosis on a gluten-free diet (GFD), compared to non-celiac osteoporotic patients. Fifteen celiac patients with osteoporosis and control subjects of the same age, sex, BMI, and fragility fractures were enrolled. At baseline, each patient underwent biochemical tests, spine X-ray, and DXA measurements, and the Charlson Comorbidity Index (CCI) was computed. At each visit (every 2 years ± 6 months), with a follow-up of 4 years, any adverse events or new clinical fractures, DXA measurements, and CCI were recorded. In celiac patients, a statistically significant median delta increase in total hip *T*-score of 0.11 compared to baseline was observed (ANOVA *p*< 0.05), while in the control group, it was 0.07 (ANOVA *p* < 0.05), with no difference between groups. New fractures occurred in the celiac group in five patients during the follow-up, and in two patients in the control group (*p* = 0.38). No adverse events occurred during follow-up. In celiac patients with osteoporosis on GFD, denosumab, with up to 4 years of follow-up, increased hip BMD without adverse events.

## Introduction

One of the most common systemic autoimmune diseases, affecting 1% of the world population, is celiac disease, a disorder characterized by small intestinal inflammation triggered by gluten ingestion in genetically predisposed individuals [1]. The symptoms of celiac disease are varied, mainly related to malabsorption, and osteoporosis is a well-recognized complication in these patients that may lead to fragility fractures [2–4].

Not only do patients with celiac disease have lower bone mineral density (BMD) compared to age- and sex-matched controls, but also the bone quality is affected with a significant impairment in bone microarchitecture as shown with high-resolution peripheral computed tomography studies [5, 6].

Two meta-analyses showed a 30% increased risk of fracture in celiac subjects for fracture at any site and a 69% increased risk for hip fracture [2, 3]. A recent Danish nationwide cohort study confirms these data in celiac patients with an HR for any fracture of 1.27 (95% CI 1.18–1.36) [4].

The etiology of bone fragility in this population is multifactorial: malabsorption leads to secondary hyperparathyroidism, but also chronic inflammation and endocrinological disorders may affect bone health [7].

BMD improvement with a gluten-free diet (GFD), which is the mainstream therapy for celiac disease, has been shown by several studies, including an improvement in bone microarchitecture [8–10].

Data on the efficacy of pharmacological treatment of osteoporosis are scant [1]. The only randomized study with an intravenous bisphosphonate, zoledronate, in 15 osteopenic and osteoporotic patients, in combination with GFD, showed no significant improvement in BMD compared to a GFD alone [11]. There are no studies with denosumab, an antiresorptive drug, which is a fully human monoclonal antibody that binds the receptor activator of NFκB ligand (RANKL). Denosumab has the advantage of being administered by subcutaneous injection every 6 months, and has been recently shown to be more effective than alendronate in reducing fractures in postmenopausal women without celiac disease [12].

The aim of the present investigation was to study the long-term effect of denosumab on BMD in osteoporotic patients with celiac disease on GFD, compared to non-celiac osteoporotic patients of the same age, sex, body mass index (BMI), and fragility fractures.

## Methods

This is a case–control retrospective longitudinal study. We retrospectively analyzed the charts from 2014 to 2024 of patients with celiac disease prescribed denosumab, a calcium supplement (1000 mg/day), and cholecalciferol (800 IU/day) in the Bone Unit of Sapienza University of Rome with at least 4 years of follow-up.

Inclusion criteria were: age range (50–85 years), diagnosis of celiac disease according to guidelines, prior to or at the time of starting denosumab therapy [13] and having vertebral or femoral fractures regardless of DXA T-score, or non-vertebral, non-femoral fractures with a lumbar or femoral T-score <  − 3 SD, or in the absence of a fracture having a T-score <  − 4 SD. We chose these criteria, because they are the criteria for denosumab reimbursement in Italy. We excluded patients with a diagnosis of cancer, kidney failure, or liver failure.

We collected the following data at baseline: blood test, including hemoglobin, serum protein, calcium and creatinine, as previously described [14]. Each subject underwent standardized lateral radiographs of the thoracic and lumbar spine. After visual inspection of these radiographs by two independent experienced observers, vertebral deformity was defined when anterior, middle, or posterior height loss was 20% with respect to the adjacent vertebra, according to Genant’s method [15].

BMD of the lumbar spine (L1–L4) in the anterior–posterior projection (we excluded fractured vertebrae) and of the hip (femoral neck and total hip) was measured in each patient with DXA (QDR 4500; Hologic, Waltham, MA). The precision errors of the lumbar spine and total hip measurements were 1.3% and 1.7%, respectively. The Charlson Comorbidity Index (CCI) was also reported [16]. Treatment prior to initiation of denosumab for osteoporosis was reported for both celiac and non-celiac patients.

At each visit (every 2 years ± 6 months), any adverse event or new clinical fracture and Charlson Comorbidity Index (CCI) were recorded, and patients underwent the same DXA measurements.

We then matched the celiac patients with controls of the same age, sex, BMI, DXA *T*-score and fractures belonging to the same center and in the same period of time and we reported for controls the same biochemical and radiological examinations over time.

The study was approved by our local ethics committee (protocol number 7649) and informed consent was obtained from each patient included in the study.

## Statistical analysis

The data are expressed as median (range). Because the data distribution did not follow a Gaussian curve, nonparametric tests were carried out. Variations between the various times considered were tested using the Friedman test and the Wilcoxon test for paired ranks. The Spearman test was used to test the association between parameters. Results were considered significant at *p* < 0.05. Statistical elaborations were carried out using the SPSS program (release 21; SPSS, Inc., Chicago, IL). Due to the absence of studies with denosumab in the celiac population, we took as reference th e only study with an antiresorptive drug (zoledronate), which after 1 year with a sample of 15 celiac patients showed a significant BMD increase.

## Results

We enrolled 22 celiac patients, but 7 were lost in follow-up, due to the COVID pandemic, thus 15 patients were finally enrolled: 13 of whom were females and 2 were males. The median age of the patients was 78 years (range 52–86 years) and the median BMI was 23.4 kg/m^2^ (range 19.5–29.5  kg/m^2^) (Table 1). All the women were postmenopausal when enrolled. All patients followed a GFD and had no symptoms or biochemical findings of malabsorption. The median follow-up time was at least 4 years and expressed in months was not significantly different in the celiac group compared to controls (48 months, range 42–54 vs 48, range 42–56 months, *p* > 0.05).

**Table 1:** Anthropometric, biochemical parameters, bone mineral density values in patients with celiac disease and controls. Results are presented as median (range)

	Celiac patients	Controls	*p* values
Age (years)	78 (range 52, 86)	79 (range 53, 84)	ns
Female/Male (number of patients)	13/2	13/2	ns
Years at menopause	50 (range 40, 54)	48.5 (range 39, 53)	ns
Body mass index (kg/m^2^)	23.4 (range 19.5, 29.5)	24.8 (range 19.1, 29.7)	ns
Hemoglobin (g/dL)	13.8 (range 12.1, 15.3)	13.8 (range 12.1, 15.8)	ns
Creatinine (mg/dL)	0.7 (range 0.5, 1.3)	0.84 (range 0.6, 1.2)	ns
Calcium (mg/dL)	9.85 (range 9.1, 10.3)	9.7 (range 9.3, 10.6)	ns
Protein (g/dL)	7.4 (range 6.5, 8)	7.3 (range 6.6, 8.5)	ns
*T*-score L1-L4 BMD (SD)	− 3.1 (range − 0.8, − 5.4)	−2.9 (range − 1.7, − 4)	ns
*T*-score Neck BMD (SD)	− 2.4 (range − 2, − 3.7)	−2.4 (range − 1, − 3.6)	ns
*T*-score Total BMD (SD)	− 2 (range − 0.4, − 3.7)	− 2.3 (range − 0.8, − 2.7)	ns
CCI	1 (range 0,6)	1 (range 0,4)	ns

*BMD*   Bone Mineral Density, *L*_*1*_*-L*_*4*_   lumbar spine, *CCI* Charlson comorbidity index, *Ns*   not significant

In the sample studied, the median time since the diagnosis of celiac disease was 11 years when enrolled (range 0–30 years); for 4 patients, the diagnosis of osteoporosis was prior to that of celiac disease, for 3 patients, the diagnosis was simultaneous, and for the remaining 8, the diagnosis of celiac disease was prior to that of osteoporosis. Based on the anamnesis of the patients, the diagnosis of celiac disease was made on the basis of gastrointestinal symptoms or in the presence of malabsorption in every patient followed by appropriate biochemical assessment. The diagnosis of celiac disease was made in 53% of the patients before the peak bone mass age. After the diagnosis of celiac disease and during the study period the patients reported a good compliance to GFD without gastrointestinal symptoms or signs of malabsorption.

Twelve celiac patients, before starting denosumab, were previously treated for osteoporosis: 10 patients with bisphosphonates, 1 patient with strontium ranelate, 1 patient with bazedoxifene. The median duration of previous therapy with bisphosphonates was 4.8 years (range 2–8). In addition, in the control group nine patients were previously treated with bisphosphonates: median 5 years (range 2–10), a period of time not statistically different compared to celiac subjects (*p* > 0.05).

There was no difference between celiac patients and controls as regards the clinical-laboratory and densitometric parameters, as reported in Table 1.

Before starting denosumab, 14 out of 15 celiac patients had fractures, 9 patients had vertebral fractures, while six patients had peripheral fractures of which 4 were Colles fractures. Finally, 9 patients had more than one fracture. The number of fractures recorded was not statistically significant compared to the controls (Table 2).

**Table 2:** Number of patients with fractures at baseline and at the end of the study period in celiac and control subjects

	Celiac		Controls	
	Baseline	New at the end of the study	Baseline	New at the end of the study
Vertebral	9	1	10	0
Femoral	0	0	1	0
Colles	4	2	4	1
Non Vertebral-Non Femoral	6	2	6	1
Fractures > 1	9	4	9	2

Since celiac disease is an autoimmune disease, an association with other autoimmune diseases was observed in our sample, such as: Hashimoto’s thyroiditis, sarcoidosis, and primary biliary cholangitis; however, CCI was not statistically significantly different from the control group (Table 1).

During the study period, we did not find differences in CCI over time between and inter groups (all *p* > 0.05, data not shown).

Denosumab increased lumbar spine *T*-score in both groups; however, the difference in each group over time and between groups was not statistically significant, as shown in Table 3. The median increase in delta *T*-score lumbar spine in the celiac group at the end of the study was 0.12 (range −0.4 to 0.48) although not statistically significant compared to controls: 0.16 (range −0.3 to −0.69), *p* > 0.05.

**Table 3:** Data are presented as median (range) of the Delta (**Δ)*** T*-score of L1-L4 and total hip in celiac and controls subjects

	Δ T- Score 0–2		Δ T- Score 2–4		Δ T- Score 0–4	
	Celiac	Controls	Celiac	Controls	Celiac	Controls
L1-L4	0.06 (range − 0.69, 0.4)	0.15 (range 0.1, 0.22)	0.06 (range − 1.3, 0.57)	0.05 (range − 0.66, 0,22)	0.12 (range − 0.4, 0.48)	0.16 (range − 0.3, 0.69)
Total hip	0.08 (range − 0.5, 0.4)	0.06 (range − 0.10, 0.29)	0.11 (range − 0.20, 0.40)	0 (range − 0.21, 0.1)	0.11* (range − 0.25, − 0.48)	0.07* (range − 0.05, 0.33)

^*^ANOVA p < 0.05 in each group over time

The median delta total hip *T*-score in celiac patients, between visits, was statistically significant with a trend of increasing from 0.08(range −0.5 to 0.40) at the second visit up to 0.11(range −0.20 to 0.40) at the third visit, reaching 0.11(range 0.25–0.48) compared to baseline (ANOVA *p* < 0.05). In addition, in the control group we found a median increase from baseline to the end of 0.07(range −0.05 to 0.33) (ANOVA *p* < 0.05). There was no difference between groups in the median delta increase of total hip *T*-score (Table 3).

In the celiac group, we observed a positive association between baseline lumbar *T*-score and BMI (*r* = 0.66, *p* < 0.05), but not in the control group. Baseline hemoglobin value in the celiac patients was positively associated with the delta increase in *T*-score at the total hip after 2 years of denosumab treatment, but not in the control group (*r* = 0.64, *p* < 0.05). The Charlson comorbidity index at the second visit was negatively associated with the delta increase in *T*-score at the total hip after 2 years of treatment (*r* =−0.60, *p* < 0.05) in the celiac group.

In the control group delta increase *T*-score at total hip at the second visit was also negatively associated with Charlson comorbidity index at the first visit (*r* =−0.63, p < 0.05) as well as Charlson comorbidity index at the second visit (*r* =−0.60, *p* < 0.05).

In the celiac group, there was a significant association between the years of gluten-free diet and the final absolute achievement of the lumbar *T*-score (*r* = 0.59, *p* < 0.05).

New clinical fragility fractures occurred** i**n the celiac group in five patients during the follow-up, and in two patients in the control group (*p* = 0.38), as reported in Table 2.

No adverse events occurred during follow-up in both groups.

## Discussion

This is the first case–control study on the long-term use of denosumab in patients with celiac disease and osteoporosis.

Moreover, we matched the controls not only for sex, age and BMI, but also for DXA T-scores and fractures, in order to reduce bias. Overall, the sample studied is at high risk of fracture due to the high prevalence of fractures when enrolled.

Interestingly, both groups had a significant increase at the femoral site with denosumab and also at the lumbar spine, which was not significant probably due to the presence of multiple vertebral fractures and also due to the small number of patients. We excluded the fractured vertebrae from the BMD measurement and this may explain the small increase of bone density observed at the lumbar spine.

This small number of patients is due to loss to follow-up that we experienced, as with every chronic disease, during the years of the COVID pandemic [17].

It should be noted that the majority of patients in both groups had a previous treatment with bisphosphonates, thus we should consider that the time, since the beginning of antiresorptive therapy is approximately 10 years, 5–6 of bisphosphonate and at least 4 of denosumab. In this context, we did not observe any adverse events, such as osteonecrosis of the jaw or atypical femoral fractures. Although they are very rare adverse events, they scare patients, also due to the fact that they are frequently reported by the media [18, 19].

In the management of secondary osteoporosis, the treatment of the underlying disease is of utmost importance [20]. Our celiac patients were on GFD, and indeed, BMI values reflect the previous compliance to GFD and have a positive association with lumbar *T*-score at baseline. Moreover, following a GFD over a long period was positively associated with the final achievement of lumbar spine *T*-score after 4 years of denosumab. Only in the celiac group, hemoglobin was positively associated with delta increase of total hip T-score, showing the importance of the association of hemoglobin levels and bone health [14]. Hemoglobin reflects also the previous compliance to GFD in celiac patients. Regardless of the presence of celiac disease, it should be noted that the burden of disease reported as CCI was negatively associated with delta hip *T*-score in both groups.

Denosumab had some advantages. Because of its subcutaneous administration, it is suitable for those who have malabsorption, although none of our patients had biochemical sign of malabsorption. Because denosumab is administered twice in 12 months, this can increase compliance to the therapy. The efficacy of denosumab in reducing the risk of fracture is well-known, although the possible differences in the risk of fracture reduction between different skeletal sites are poorly understood (12). Moreover, one study in celiac patients reported that the osteoprotegerin/RANKL ratio was significantly lower in celiac patients than in controls and was positively correlated with BMD at the spine [21]. Thus, denosumab could also be effective, as an antibody against RANKL, acting in this unbalanced bone pathway in celiac disease.

The only study, in the literature, that tested another antiresorptive drug’s efficacy in celiac disease was carried out in a very different population compared to our patients. Indeed, in this previous study, 15 celiac patients with T-score < 1 SD (both osteoporotic and osteopenic) with a mean age of 25.3 ± 9.1 years were included [11]. After 1 year, zoledronate increased *T*-score at the lumbar spine of approximately 35.9%; however, the difference was not statistically significant compared to 13 celiac patients on GFD alone [11]. The difference in mean age, BMD, and the absence of fractures in the group treated with zoledronate compared to our patients does not make a comparison with this study possible.

This study has a few limitations. The small sample size probably does not allow us to observe differences compared to the control groups. In the follow-up, we have densitometric data and reported clinical fractures, but we did not have a morphometric evaluation for vertebral fractures.

In conclusion, this study for the first time showed an improvement, in celiac patients with osteoporosis, of BMD at the femoral site following denosumab, up to 4 years, without an adverse event. As our sample is at very high risk of fracture in the future, and with patients whose age at diagnosis of celiac disease is on average young, our results cannot be extended to the entire population of celiac patients. Further studies with a larger number of subjects, including males, are needed in order to better target osteoporosis treatment in the celiac population.

## Data Availability

Not applicable.
